# VPS34 regulates TSC1/TSC2 heterodimer to mediate RheB and mTORC1/S6K1 activation and cellular transformation

**DOI:** 10.18632/oncotarget.10469

**Published:** 2016-07-07

**Authors:** Nishant Mohan, Yi Shen, Milos Dokmanovic, Yukinori Endo, Dianne S. Hirsch, Wen Jin Wu

**Affiliations:** ^1^ Division of Biotechnology Review and Research I, Office of Biotechnology Products, Office of Pharmaceutical Quality, Center for Drug Evaluation and Research, U.S. Food and Drug Administration, Silver Spring, 20993, Maryland, USA

**Keywords:** VPS34, TSC1/TSC2, RheB, mTORC1/S6K1, cellular transformation

## Abstract

VPS34 is reported to activate S6K1 and is implicated in regulating cell growth, the mechanisms of which remain elusive. Here, we describe novel mechanisms by which VPS34 upregulates mTOR/S6K1 activity via downregulating TSC2 protein and activating RheB activity. Specifically, upregulation of VPS34 lipid kinase increases local production of ptdins(3)p in the plasma membrane, which recruits PIKFYVE, a FYVE domain containing protein, to ptdins(3)p enriched regions of the plasma membrane, where VPS34 forms a protein complex with PIKFYVE and TSC1. This in turn disengages TSC2 from the TSC1/TSC2 heterodimer, leading to TSC2 ubiquitination and degradation. Downregulation of TSC2 promotes the activation of RheB and mTOR/S6K1. When VPS34 lipid kinase activity is increased by introduction of an H868R mutation, ptdins(3)p production at the plasma membrane is dramatically increased, which recruits more PIKFYVE and TSC1 molecules to the plasma membrane. This results in the enhanced TSC2 ubiquitination and degradation, and subsequent activation of RheB and mTORC1/S6K1, leading to oncogenic transformation. The role played by VPS34 in regulating mTOR/S6K1 activity and cellular transformation is underscored by the fact that the VPS34 kinase dead mutant blocks VPS34-induced recruitment of PIKFYVE and TSC1 to the plasma membrane. This study provides mechanistic insight into the cellular function of VPS34 in regulating oncogenic transformation and important indications for identifying VPS34 specific mutations in human cancers.

## INTRODUCTION

The mammalian target of rapamycin complex 1 (mTORC1) has been established as a critical molecule in the regulation of protein translation, cell cycle progression and cell proliferation [1–4]. It is believed that deregulation of mTORC1 signaling in tumors is due to either loss of function of upstream tumor suppressor proteins or activating mutations within oncogenes that upregulate mTOR pathway [5].

*TSC1* and *TSC2* are the tumor-suppressor genes mutated in tumor syndrome TSC (tuberous sclerosis complex). Over the past decade, their gene products, TSC1/TSC2 heterodimer, have emerged as a critical integrator of growth factor, nutrient and stress signals to control protein synthesis, cell growth and other cellular processes [6]. It is now recognized that the primary function of the TSC1/TSC2 heterodimer is as a critical negative regulator of mTORC1 activation, where TSC2 exhibits a selective GTPase-activating protein (GAP) activity toward the small GTPase, RheB (Ras homologue enriched in brain). RheB is an upstream positive regulator of mTORC1 [7–9]. Overexpression of RheB in mammalian cells leads to the activation of mTORC1 in the absence of growth factors [10]. While the GAP domain of TSC2 contains the tumor suppressor activity, studies indicate that TSC1/TSC2 function primarily as a protein complex, and that TSC1 is required for the stabilization of TSC2 and prevents TSC2 ubiquitination by HERC1 ubiquitin ligase and subsequent degradation [11–13]. The abundance of data suggest that Akt phosphorylates TSC2, which reduces the inhibitory effects of TSC1/TSC2 heterodimer on mTORC1 resulting in the activation of mTOR [10]. However, the precise mechanism by which Akt phosphorylation affects the function of TSC1/TSC2 heterodimer is not clear [14]. Moreover, no difference in GAP activity towards recombinant RheB is detected between wild type TSC2 and phosphorylated TSC2 [6].

Vacuolar protein sorting 34 (VPS34), class III PI3K, mediates nutrient signaling to mTORC1, leading to the activation of S6 Kinase 1 (S6K1) and regulation of protein synthesis [15–19]. Inhibition of VPS34 by overexpression of FYVE domains, which binds to and sequesters ptdins(3)p, microinjection of inhibitory antibodies or siRNA-mediated knockdown of VPS34 expression blocks insulin-stimulated phosphorylation of both S6K1 and 4EBP1 [15, 16]. Conversely, overexpression of VPS34 activates S6K1 in the absence of insulin stimulation [19]. We recently demonstrated that insulin was able to spatially regulate VPS34 activity to produce ptdins(3)p at the plasma membrane to mediate co-localization between VPS34 and phosphatidylinositol 3-phosphate 5-kinase (PIKFYVE), a FYVE domain containing protein, at the plasma membrane and the activation of S6K1 [20]. However, the mechanisms by which VPS34 regulates S6K1 remain elusive.

Class I PI3K plays critical roles in cell growth and its tumorigenic activity is activated by somatic point mutations [21, 22]. p110α of class I PI3K is encoded by the *PIK3CA* gene [21]. A high frequency of somatic point mutations in the *PIK3CA* gene has been found in human cancers [22–26]. Cancer-specific mutations frequently occur in several “hotspots” of the helical (E542 and E545) and kinase (H1047) domains of PI3K p110α [22–26]. E542 and E545 are often substituted with lysine in the colon and brain tumors, whereas H1047 is frequently substituted with arginine in tumors of breast, colon, and brain [22–24]. The mutant proteins display higher lipid kinase activity as compared with wild-type p110α, suggesting that these mutations induce enzymatic gain of function. Furthermore, the cancer-specific mutations are oncogenic both *in vitro* and *in vivo* [27, 28]. While cancer-specific mutations have not been found in VPS34, a study reported that VPS34 gene expression was increased 2.5 times in epithelial dysplasia and 11 times in tongue cancer tissues as compared with normal tissues [29]. We reported that the protein levels of VPS34 were upregulated in highly tumorigenic breast cancer cell lines (MDA-MB-231, MDA-MB-468, and SKBR3) as compared with relatively low tumorigenic breast cancer cell lines (MCF-7 and T47D) and a normal cell line (MCF-10A), and that VPS34 lipid kinase activity is required for Src-induced cellular transformation [30]. However, the role of VPS34 in the regulation of cell growth and transformation has not been well studied. In this study, we find that VPS34 binds to TSC1, resulting in TSC2 ubiquitination and degradation. This in turn activates RheB and mTOR/S6K1 to mediate cellular transformation and tumor formation.

## RESULTS

### Introduction of an H868R point mutation in VPS34 kinase domain increases its lipid kinase activity

**Figure 1:**
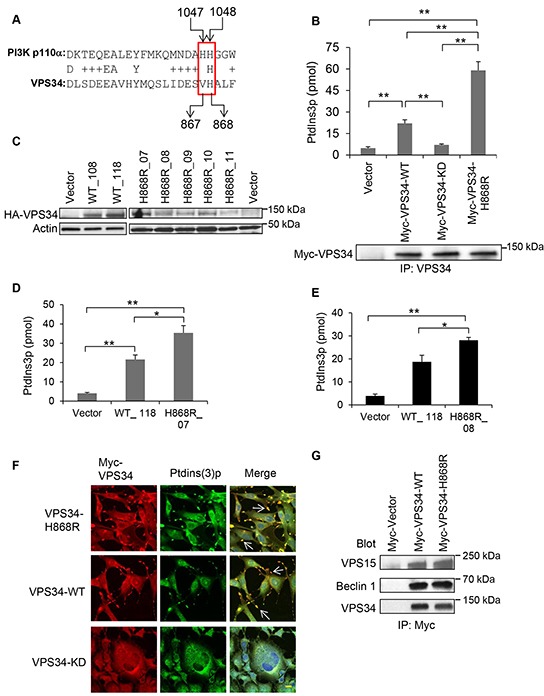
VPS34-H868R exhibits upregulated lipid kinase activity **A.** Sequence alignment between human VPS34 and p110α was performed using the NCBI blast alignment tool. **B.** COS-7 cells were transiently transfected with plasmids encoding the indicated proteins. Anti-Myc antibody was used to immunoprecipitate Myc-tagged proteins. Immunoprecipitates were subjected to an *in vitro* lipid kinase assay. Ptdins(3)p production (pmol) for each enzymatic reaction was determined by interpolation from the standard curve. Bar graph is representative of two or more experiments and presented in the form of mean ± SEM (**, p < 0.01). The protein levels of Myc-tagged VPS34 proteins immunoprecipitated from whole cell lysates (WCL) were detected by Western blot analysis using anti-Myc antibody. **C.** NIH3T3 cells stably expressing HA-VPS34-WT, HA-VPS34-H868R, or empty vector were generated by co-transfection of the indicated pJ4-HA-VPS34 constructs with pcDNA3 empty vector. Stable expression of HA-VPS34-WT and HA-VPS34-H868R clones were determined by Western blot analysis using anti-HA antibody. Actin Western blot analysis of WCL was done to control for equal loading. From now on, actin Western blot analysis was performed to confirm equal loading. **D.** *In vitro* lipid kinase assay was performed with cell lines stably expressing Vector, HA-VPS34-WT (clone WT_118) and HA-VPS34-H868R (clone H868R_07) after immunoprecipitating the protein using anti-HA antibody. Ptdins(3)p production (pmol) for each enzymatic reaction was determined by interpolation from the standard curve. Bar graph is representative of two or more experiments and presented in the form of mean ± SEM (*, p < 0.05; **, p < 0.01). **E.** The methods were the same as described in Figure 1D except that the *in vitro* lipid kinase experiment was done using a different HA-VPS34-H868R clone (H868R_08). **F.** NIH3T3 cells were seeded overnight in fibronectin-coated chamber slides and then transfected with Myc-tagged VPS34 proteins. Cells were immunostained for Myc-VPS34 (red) and ptdins(3)p (green) Bar: 20 μm. Images were captured on Zeiss LSM-510 Meta microscope. Nuclear DAPI staining was shown in blue color in merged images. The colocalization of Myc-VPS34 and ptdins(3)p was shown in yellow color and indicated by arrows in merged images. Bar: 20 μm. **G.** COS-7 cells were transfected with pcDNA3-Myc-Vector, pcDNA3-Myc-VPS34-WT, pcDNA3-Myc-VPS34-H868R. 48h post transfection, the Myc-tagged VPS34 proteins were immunoprecipitated from WCL using an anti-Myc antibody. VPS15 and Beclin 1 were detected by Western blot analysis; immunoprecipitated Myc-VPS34 was detected by blotting with anti-VPS34 antibody.

We searched for VPS34 mutations with potential gain of lipid kinase activity by performing a sequence alignment of VPS34 and p110α of class I PI3K. We observed that while the H1047 hot spot of p110α was not conserved in VPS34 (V867), the site H1048 of p110α was conserved with VPS34 (H868) (Figure 1A). We generated a VPS34 point mutant by substituting an arginine at H868 (VPS34-H868R). To detect the effects of this mutant on VPS34 lipid kinase activity, COS7 cells were transiently transfected with either the Vector or the plasmids encoding VPS34 wild type (Myc-VPS34-WT), VPS34 lipid kinase dead (Myc-VPS34-KD) [20], or VPS34-H868R (Myc-VPS34-H868R). Figure 1B showed that the lipid kinase activity of VPS34-H868R was significantly increased as compared with that of Vector control (p < 0.01), VPS34-WT (p < 0.01), and VPS34-KD (p < 0.01). As expected, the basal level of lipid kinase activity of VPS34-WT was significantly higher than that of Vector control (p < 0.01) and VPS34-KD (p < 0.01), consistent with our previous report [20]. To substantiate the enhanced lipid kinase activity of VPS34-H868R in transiently transfected cells, NIH3T3 cell lines stably expressing Vector, VPS34-WT and VPS34-H868R were generated as previously reported [30, 31], and the expression levels were monitored in these cell clones (Figure 1C). As shown in Figure 1D and 1E, the lipid kinase activities obtained from the indicated cell lines (WT_118, H868R_07 and H868R_08) were consistent with data described in Figure 1B. Figure 1F demonstrated that NIH3T3 cells transiently expressing Myc-VPS34-H868R exhibited abundant co-localization of VPS34 and ptdins(3)p at membrane ruffles. VPS34 and ptdins(3)p were also co-localized at the membrane ruffles in the cells transiently expressing Myc-VPS34-WT, but to a less extent as compared with the cells expressing Myc-VPS34-H868R. Myc-VPS34-KD transfected cells did not display co-localization of VPS34 and ptdins(3)p at the plasma membrane, nor were membrane ruffles observed (Figure 1F). Pearson's colocalization coefficient values were calculated by ZEN software from Carl Zeiss Micro Imaging. These values were counted on membrane ruffles of approximately 20 cells from 5 different fields in each group and presented in form of mean ± STDEV. The colocalization coefficient values of Myc-VPS34 and ptdins(3)p were 0.61 ± 0.12 in VPS34-H868R 0.54 ± 0.11 in VPS34-WT and −0.02 ± 0.19 for VPS34-KD group. Figure 1G showed that VPS34-H868R did not interfere with the binding of VPS34 to Beclin 1 or VPS15, suggesting that the increased lipid kinase activity of VPS34-H868R is independent of VPS34 binding to Beclin 1 and VPS15.

### VPS34 activates mTORC1/S6K1 activity, leading to an increase in cell size

**Figure 2:**
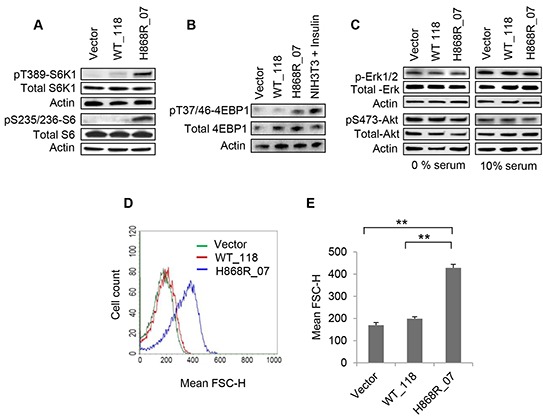
VPS34 activates mTORC1/S6K1, leading to the increase in cell size (referring to Figure 1C for the clones stably expressing vector, VPS34-WT and VPS34-H868R) **A.** WCL obtained from indicated stable cell clones, vector, WT_118 and H868R_07 was subjected to western blotting to monitor the phosphorylated and total expression levels of S6K1. **B.** Stable Vector, WT_118 and H868R_07 cells were assessed for phosphorylation of 4EBP1 at T37/46 position by Western blot analysis. Insulin-treated (100 nM for 30 min) NIH3T3 cells were used as a positive control for 4EBP1 phosphorylation. The membrane was stripped and re-probed for total 4EBP1. **C.** Phosphorylation of Erk1/2 and Akt (S473) were detected in stable cell clones as indicated in 1% and 10% serum containing media. Membranes was stripped and re-blotted with total Erk1/2 and Akt proteins. **D.** The relative cell size of the indicated stable clones was analyzed using a BD FACSCalibur flow cytometer to determine the mean FSC-H of cells for the measurement of relative cell size. **E.** Quantitative data of flow cytometric cell size were performed from two or more independent experiments and presented in the form of bar graph (**, p < 0.01).

We next tested whether upregulation of VPS34 affected mTOR activity and its downstream signaling pathways. Figure 2A showed that the activity of mTORC1/S6K1 as measured by T389-S6K1 and S235/236-S6 phosphorylation was dramatically increased in H868R_07 cells as compared with that of WT_118 and Vector control cells. A modest activation of mTORC1/S6K1 was observed in WT_118 cells compared with Vector control cells (Figure 2A). Figure 2B showed that 4EBP1 phosphorylation at T37/T46 was increased in H868R_07 cells as compared with WT_118 and Vector control cells. These data indicated that the ability of VPS34 to upregulate mTORC1 and its downstream signaling correlated to its kinase activity. Figure 2C showed that the activities of Akt and Erk1/2 were not changed in WT_118 and H868R_07 cells as compared with Vector cells, indicating that VPS34-mediated activation of mTORC1/S6K1 signaling was independent of class I PI3K/Akt pathway. The increase in S6K1 activity in H868R_07 cells prompted us to investigate the regulation of cell size. Quantitative analysis of flow cytometric data unveiled that H868R_07 cells have manifested a remarkable rightward shift in FSC-H histogram denoting that these cells were significantly bigger in dimensions than WT_118 (p < 0.01) and Vector cells (p < 0.01) (Figure 2D and 2E).

### VPS34 binds to TSC1, but not TSC2

**Figure 3:**
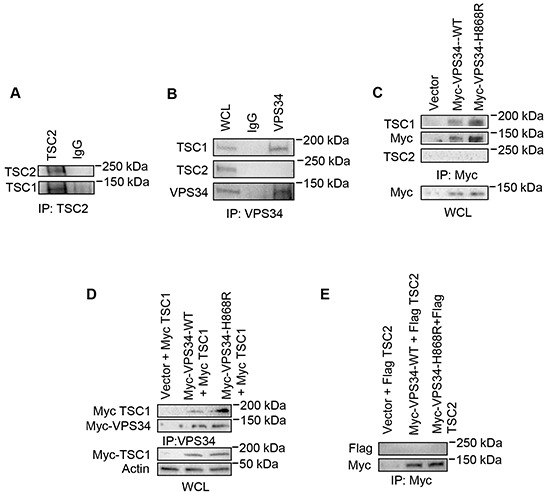
VPS34 binds to TSC1 but not TSC2 **A.** Endogenous TSC1 was co-immunoprecipitated from NIH3T3 cell WCL using anti-TSC2 antibody. **B.** Endogenous TSC1, but not TSC2, was co-immunoprecipitated from NIH3T3 WCL using anti-VPS34 antibody. Endogenous TSC1, TSC2 and VPS34 in WCL were detected using their corresponding antibodies. **C.** NIH3T3 cells were transiently transfected with pcDNA3-Myc-Vector, pcDNA3-Myc-VPS34-WT, or pcDNA3-Myc-VPS34-H868R. Anti-Myc antibody was used to immunoprecipitate Myc-tagged proteins. Endogenous TSC1 or TSC2 in the immunoprecipitates was detected by Western blot analysis using anti-TSC1 or anti-TSC2 antibody. Expression of Myc-VPS34 proteins in WCL was detected using anti-Myc antibody. **D.** COS-7 cells were transiently co-transfected with the indicated plasmids. VPS34 proteins were immunoprecipitated using anti-VPS34 antibody. Myc-TSC1 and Myc-VPS34 proteins in immunoprecipitates were detected using anti-Myc antibody. Myc-TSC1 expression in WCL was detected using anti-Myc antibody. **E.** COS7 cells were transiently co-transfected with plasmids encoding myc-VPS34-WT or Myc-VPS34-H868R plus Flag-TSC2 plasmids. Myc-tagged VPS34 proteins were immunoprecipitated used anti-Myc antibody. Immunoprecipitates were probed with anti-Flag antibody for TSC2 and then reprobed with anti-Myc antibody for VPS34 proteins.

We next investigated the relationship between VPS34 and TSC1/TSC2 heterodimer. Figure 3A showed that endogenous TSC1 was found in TSC2 immunoprecipitates in NIH3T3 cells. Endogenous TSC1, but not endogenous TSC2, was co-immunoprecipitated with endogenous VPS34 (Figure 3B) or transiently expressed Myc-VPS34 in NIH3T3 cells (Figure 3C). This observation was further confirmed when Myc-TSC1 or Flag-TSC2 was transiently co-expressed with Myc-VPS34 in COS7 cells. As shown in Figure 3D, Myc-TSC1 associated with Myc-VPS34 with an enhanced binding toward Myc-VPS34-H868R mutant as compared with Myc-VPS34-WT. Figure 3E showed that the transiently expressed Flag-TSC2 was not found in Myc-VPS34 immunoprecipitates.

### VPS34 co-localizes with TSC1 and PIKFYVE at membrane ruffles

**Figure 4:**
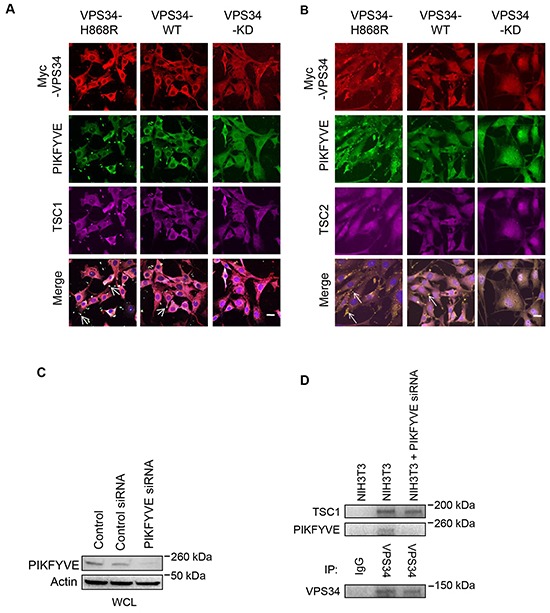
VPS34 co-localizes with TSC1 and PIKFYVE, but not with TSC2 at the plasma membrane **A.** NIH3T3 cells were transfected with Myc-tagged VPS34 proteins. Cells were immunostained for Myc-VPS34 (red), PIKFYVE (green) and TSC1 (magenta). Bar: 20 μm. The white color indicates colocalization of VPS34, PIKFYVE, and TSC1 (Arrows in merged images). **B.** Experimental procedures were essentially the same as that described in (A) except cells were immunostained for TSC2 (magenta). Yellow color indicates colocalization of VPS34 and PIKFYVE (arrows in merged images). Bar: 20 μm. All images in Figure 4 were captured on Zeiss LSM-510 Meta microscope. **C.** NIH3T3 cells were transfected with control and PIKFYVE siRNA, and Western blot analysis was performed to show PIKFYVE knockdown. **D.** NIH3T3 cells were transfected with PIKFYVE siRNA for 48h or left un-transfected. Indicated WCL were subjected to immunoprecipitation using anti-VPS34 antibody or control IgG. The levels of TSC1, PIKFYVE and VPS34 were detected by Western blotting in immunoprecipitated samples.

It has been reported that the FYVE domain containing protein, PIKFYVE, specifically recognizes ptdins(3)p and targets cytosolic proteins to ptdins(3)p-enriched membranes and mediates mTOR activation [32, 33]. We used PIKFYVE as a marker for ptdins(3)p production and membrane localization of VPS34 and observed an enhanced co-localization of Myc-VPS34-H868R and PIKFYVE at the plasma membrane as compared with that of Myc-VPS34-WT and PIKFYVE (Figure 4A, compare with left panels with middle panels). This may be the consequence of increased ptdins(3)p production by VPS34-H868R mutant at plasma membrane, which recruited more PIKFYVE to the plasma membrane. Consistent with findings described in Figure 3, TSC1, but not TSC2, co-localized with VPS34 and PIKFYVE at the plasma membrane with enhanced co-localization of TSC1 with VPS34-H868R and PIKFYVE as compared to that VPS34_WT and PIKFYVE (Figure 4A and 4B). Furthermore, the co-localization of VPS34 with PIKFYVE and TSC1 at the plasma membrane was interrupted when VPS34 lipid kinase is impaired (Figure 4A right panels), indicating that VPS34 kinase activity is essential for VPS34 co-localization with PIKFYVE and TSC1 at the plasma membrane. Pearson's correlation coefficient was calculated using ZEN confocal software after counting approximately 20 cells in 5 different fields from each group and presented in the form of mean ± STDEV. The colocalization coefficient values of Myc-VPS34 and PIKFYVE were 0.62 ± 0.15 in VPS34-H868R, 0.56 ± 0.13, in VPS34-WT and −0.003 ± 0.21 in VPS34-KD group. The colocalization coefficient values of Myc-VPS34 and TSC1 were 0.66 ± 0.13 in VPS34-H868R, 0.53 ± 0.12 in VPS34-WT and −0.007 ± 0.08 in VPS34-KD group. The colocalization coefficient values of PIKFYVE and TSC1 were 0.63 ± 0.14 in VPS34-H868R, 0.54 ± 0.13 in VPS34-WT and −0.02 ± 0.09 in VPS34-KD group. Additionally, we explored whether PIKFYVE knockdown could modulate VPS34/PIKFYVE/TSC1 association. PIKFYVE knockdown was accomplished by using PIKFYVE specfic siRNA (Figure 4C). Immunoprecipitation data shown in Figure 4D suggest that VPS34/PIKFYVE/TSC1 binding occurs at endogenous level in NIH3T3 cells and all three proteins exist in a complex. However, when PIKFYVE was knockdown using RNAi, VPS34 still associates with TSC1, implying that VPS34/TSC1 binding is independent of PIKFYVE.

### Binding of VPS34 to TSC1 mediates TSC2 ubiquitination and TSC1/TSC2 degradation, leading to RheB activation

**Figure 5:**
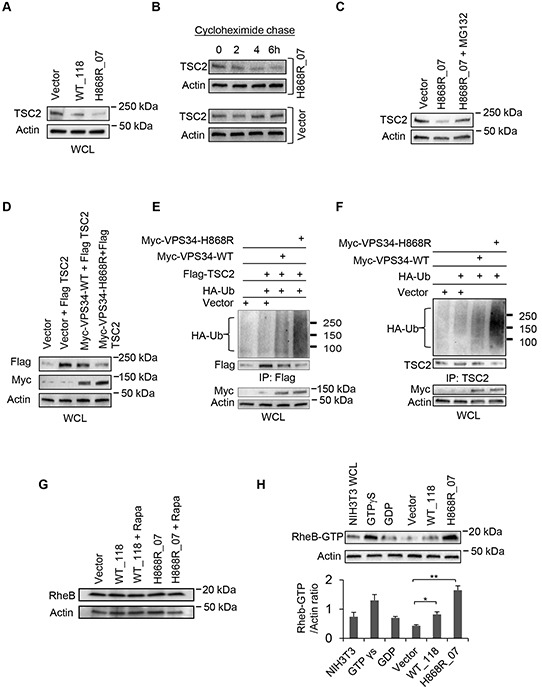
Binding of VPS34 to TSC1 mediates TSC2 ubiquitination and degradation, and the activation of RheB **A.** Western blot analysis was performed to detect the endogenous levels of TSC2 in Vector, WT_118 and H868R_07 stable lines using anti-TSC2 antibodies. **B.** Cycloheximide chase experiment was performed to monitor TSC2 degradation. Vector and H868R_07 stable cells were incubated with cycloheximide at 50 μg/ml for indicated times. After incubation, WCL was collected and western blotting was performed to detect the levels of TSC2 using anti-TSC2 antibody. **C.** Stably H868R_07 cells were treated with MG132 at 5 μM for 24h or left untreated. WCL was subjected to western blot analysis to observe the levels of TSC2 protein expression. **D.** COS7 cells were transiently co-transfected with plasmids encoding the indicated proteins. 48h post-transfection, Flag-tagged TSC2 and Myc-tagged proteins in WCL were detected by Western blotting using anti-Flag antibody and anti-Myc antibody, respectively. **E.** COS7 cells were transiently co-transfected with Vector or the indicated Myc-VPS34 constructs along with Flag-TSC2 and HA-Ubiquitin (HA-Ub). 48h post-transfection, Flag-tagged TSC2 was immunoprecipitated from WCL using an anti-Flag antibody. Ubiquitinated Flag-TSC2 was detected by anti-HA antibody and immunoprecipitated Flag-TSC2 was detected by anti-Flag antibody. Myc-tagged proteins in WCL were detected using anti-Myc antibody. **F.** NIH3T3 cells were transiently co-transfected with Vector or the indicated Myc-VPS34 constructs plus HA-Ubiquitin plasmid. 48h post-transfection, endogenous TSC2 was immunoprecipitated from WCL and ubiquitinated TSC2 was detected by Western blot using anti-HA antibody. The immunoprecipitated TSC2 was detected by Western blot using anti-TSC2 antibody for assessing TSC2 degradation. The protein levels of Myc-VPS34 proteins in the WCL were detected by Western blot using anti-Myc antibody. **G.** Expression of endogenous RheB was monitored in WCL of Vector, and WT_118 and H868R_07 cells, which were treated with rapamycin (10 nM) for 2 days or left untreated, by Western blot analysis using an anti-RheB antibody. **H.** RheB activation assay was performed to assess the levels of active RheB-GTP in Vector, WT_118 and H868R_07 cell clones. The RheB-GTP was detected by Western blot analysis using a rabbit polyclonal anti-RheB-GTP antibody. Quantitative analysis of RheB-GTP and actin was determined from three independent experiments and expressed as mean ± SEM (*, p < 0.05; **, p < 0.01).

Given that TSC1 is required for the stability of TSC2 [13], we hypothesized that binding of VPS34 to TSC1 disengaged TSC1/TSC2 heterodimer, which may lead to degradation of TSC2. To test this hypothesis, we first determined the levels of TSC2 in Vector, WT_118 and H868R_07 stable cell lines. Figure 5A showed that the levels of endogenous TSC2 were reduced in VPS34-WT and VPS34-H868R expressing cells as compared to Vector with the greatest reduction in protein levels of TSC2 observed in cells expressing VPS34-H868R. To test the TSC2 stability in cells stably expressing H868R_07, we set out time course experiments by treating H868R_07 and vector cells with cycloheximide, an inhibitor of protein biosynthesis. In H868R_07 cells, the protein levels of TSC2 were reduced after 2-4h of cycloheximide treatment, however, in vector cells, no appreciable TSC2 reduction in protein levels was detected even 6h after addition of cycloheximide (Figure 5B), suggesting that the rate of TSC2 degradation is enhanced in VPS34-H868R expressing cells. Furthermore, we observed the restoration of TSC2 expression levels in stably expressing H868R_07 cells when incubated with MG132, a proteasome inhibitor (Figure 5C). Downregulation of TSC2 was also found in our transient expression system when Flag-TSC2 was co-expressed with Myc-VPS34 proteins (Figure 5D). We next tested whether binding of VPS34 to TSC1 induced TSC2 ubiquitination and degradation. Flag-tagged TSC2 and endogenous TSC2 were ubiquitinated and degraded when Myc-VPS34 proteins were transiently expressed in either COS7 (Figure 5E) or NIH3T3 cells (Figure 5F). Moreover, TSC2 ubiquitination and degradation were increased in cells expressing Myc-VPS34-H868R as compared with Myc-VPS34-WT (Figure 5E and 5F).

RheB is inactivated by GTP hydrolysis catalyzed by TSC2. We next tested whether VPS34-mediated downegulation of TSC2 resulted in activation of RheB. Figure 5G showed that the levels of RheB expression in WT_118 and H868R_07 cells untreated or treated with rapamycin (Rapa) remained unchanged as compared with Vector cells. However, active RheB (RheB-GTP) was significantly increased in H868R_07 cells as compared with Vector (Figure 5H). An increase in RheB-GTP was also observed in WT_118 cells compared with Vector cells (Figure 5H).

### VPS34 promotes cell cycle progression and stimulates DNA synthesis

**Figure 6:**
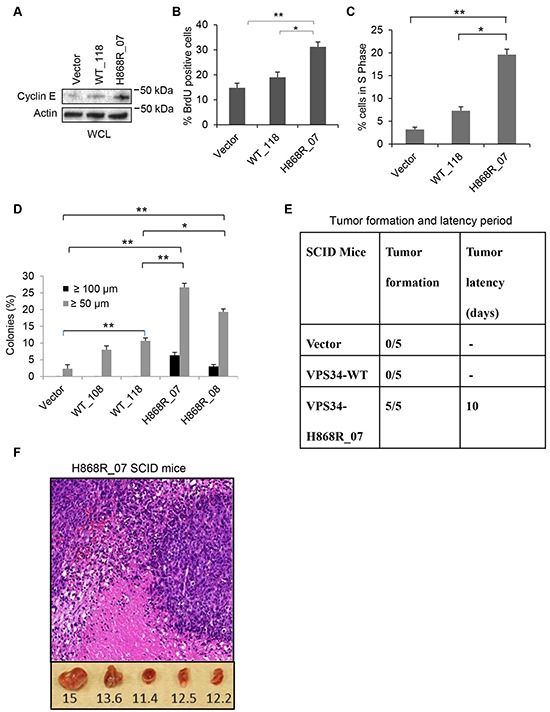
VPS34-H868R induces cell cycle progression, cellular transformation, and tumor formation in mice **A.** Cyclin E expression levels were determined in Vector, WT_118 and H868R_07 stable clones by Western blot analysis using anti-cyclin E antibody. **B.** BrdU immunofluorescence staining was done on Vector, WT_118 and H868R_07 stable cell clones. Quantitative analysis of BrdU incorporation was done by counting the BrdU-positive cells in three randomly selected microscopic fields from two or more independent experiments. Data are presented as the mean percentage of BrdU-positive cells as mean ± SEM (*, p < 0.05; **, p < 0.01). **C.** Flow cytometric cell cycle analysis of BrdU incorporation was done in Vector, WT_118 and H868R_07 cells as described in Materials and Methods. Quantitative analysis of BrdU flow cytometric cell cycle data was performed and presented as percentage of BrdU-positive cells that are present in S phase of cell cycle. (*, p < 0.05; **, p < 0.01). **D.** Vector, VPS34-WT (clones WT_108 and WT_118) and VPS34-H868R (clones H868R_07 and H868R_08) expressing cells were seeded in soft agar. Cells were fed once per week with soft agar containing 10% CS. Colonies ≥ 50 μm or ≥ 100 μm were quantified on day 21 post-seeding. Data are the mean ± SEM of two or more independent experiments run in duplicate (*, p < 0.05; **, p < 0.01). **E.** Summary of tumor formation and latency period. Tumor latency is the time between the injection and the time of detection of a tumor larger than 5 mm. **F.** Representative histopathological H&E staining of tumor tissue from SCID mice injected with H868R_07 cells (magnification, x20). Lower panel: Tumor measurements in millimeters.

Cyclin E plays a critical role in cell cycle progression from G1 to S phase. Western blot data showed that cyclin E expression was higher in H868R_07 cells than WT_118 and Vector cells (Figure 6A). Cyclin E expression was slightly increased in WT_118 cells as compared with Vector cell (Figure 6A). BrdU immunofluorescence staining exhibited a significant increase in BrdU incorporation in H868R cells as compared with that in WT_118 and Vector cells, suggesting an increase in DNA synthesis in mutant cells (Figure 6B). BrdU flow cytometric analysis of cell cycle progression revealed significant accumulation of H868R_07 cells in S phase of cell cycle in comparison to WT_118 (p < 0.05) or Vector cells (p < 0.01) (Figure 6C). A modest increase in percentage of cells in S phase was observed in WT_118 cells as compared with Vector cells (p = 0.073) (Figure 6C). Taken together, these data indicate that overexpression of VPS34 promotes cell cycle progression and that ability of VPS34 to promote cell cycle progression correlates with its lipid kinase activity.

### Upregulation of VPS34 induces cellular transformation and tumor formation in mice

To test whether overexpression of either VPS34-WT or VPS34-H868R induces cellular transformation, two VPS34-WT clones (WT_108 and WT_118) and two VPS34-H868R clones (H868R_07 and H868R_08) with different levels of VPS34 expression (Figure 1C) were randomly selected for this experiment. As shown in Figure 6D, anchorage independent growth of the cells stably expressing VPS34-WT or VPS34-H868R was significantly enhanced as compared with Vector cells [H868R_07 vs. Vector (p < 0.01); H868R_08 vs. Vector (p < 0.01); WT_118 vs. Vector (p < 0.01)]. Furthermore, the extent of anchorage independent growth correlated with the expression levels of VPS34-WT or VPS34-H868R (H868R_07 vs. H868R_08) or VPS34-WT (WT_118 vs. WT_108). Importantly, the ability of VPS34 to induce anchorage independent growth correlated with its lipid kinase activity in that the growth in soft agar was significantly increased in cells expressing VPS34-H868R as compared with cells expressing VPS34-WT [H868R_07 vs. WT_118 (p < 0.01); H868R_08 vs. WT_118 (p < 0.05)] (Figure 6D). Additionally, cells expressing VPS34-H868R (H868R_07 or H868R_08) were capable of forming colonies that were ≥ 100 μm in size. Taken together, these data indicated that the oncogenic activity of VPS34 correlated with its lipid kinase activity.

We next asked whether overexpression of VPS34-WT or VPS34-H868R induced tumor formation in mice. Subcutaneous injection of H868R_07 cells into severely compromised immune-deficient (SCID) mice resulted in the formation of solid tumors after 1-2 weeks (Figure 6E and 6F). The histopathological evaluation of tumor sections revealed the development of fibrosarcoma with infiltration of inflammatory cells and presence of necrotic cells (Figure 6F). However, WT_118 and Vector cells failed to form tumors in SCID mice (Figure 6E). These data indicate that VPS34, when its kinase activity is upregulated, is tumorigenic.

### Inhibition of mTORC1 and S6K1 diminishes VPS34-induced cellular transformation

**Figure 7:**
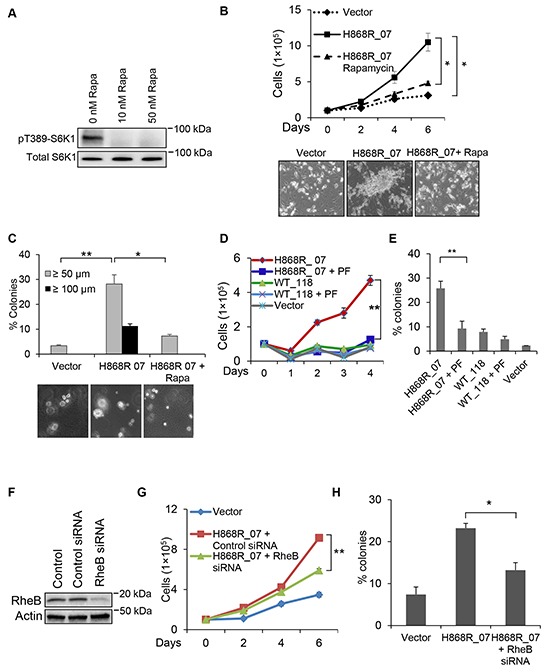
mTOR/S6K1 signaling pathway is necessary for VPS34-induced oncogenic transformation **A.** VPS34-H868R_07 cells were treated with rapamycin (Rapa) for 1h at the indicated concentrations or left untreated. The levels of pT389-S6K1 in WCL were detected by Western blot analysis using antibody directed against pT389-S6K1. **B.** Growth profiles (1% CS) of Vector cells and H868R_07 cells treated with rapamycin (10 nM) or left untreated for the indicated time. Data are representative of two or more experiments and presented in the form of mean ± STDEV (*, p < 0.05); Lower panel: phase-contrast micrographs of representative images from the indicated cell lines cultured in low serum media for six days (magnification, ×100). **C.** Experimental procedures for the soft agar assay were essentially the same as described in Figure 6D except that H868R_07 cells were treated with rapamycin (10 nM) or left untreated. Upper panel bar graph are representative of two or more experiments and presented in the form of mean ± SEM (*, p < 0.05; **, p < 0.01). Lower panel shows the phase-contrast micrographs of representative images from the indicated cell lines (magnification, ×100). Images were captured on day 21. **D.** Vector, WT_118 and H868R_07 cells were seeded in duplicate in DMEM media containing 1% CS. WT_118 and H868R_07 cells were treated with PF-4708671 (10 μM) for the indicated times or left untreated. At the indicated days, cells were harvested and counted. Line graph is representative of two or more experiments and presented in the form of mean ± SEM (**, p < 0.01). **E.** Vector, WT_118, and H868R_07 cells were seeded in 6-well plate in soft agar containing 10 μM PF or 0 μM PF. After 3 weeks, colonies were counted with criterion ≥50 μm. Bar graph is representative of two or more experiments and presented in the form of mean ± SEM (**, p < 0.01). **F.** Western blot analysis was done to show RheB knockdown in NIH3T3 cells that were transfected with either control of RheB siRNA. **G.** Vector and H868R_07 cells were seeded in duplicate in DMEM media containing 1% CS. H868R_07 cells were transfected with RheB siRNA or left non-transfected. At the indicated days, cells were harvested and counted. Line graph is representative of two or more experiments and presented in the form of mean ± SEM (**, p < 0.01). **H.** Soft agar colony formation assay for vector, H868R_07 and H868R_07 + RheB siRNA. Bar diagram is representative of two or more experiments and presented in the form of mean ± SEM (*, p < 0.05).

We next used pharmacological approaches to confirm that cell growth and transformation induced by VPS34 occurred via mTORC1/S6K1 pathway. Figure 7A showed that S6K1 phosphorylation in H868R_07 cells was completely inhibited by treatment of cells with 10 or 50 nM of rapamycin (Rapa), an mTORC1 specific inhibitor. Figure 7B showed that H868R_07 cells were capable of growing in low serum media (1% CS) and that on day 6 the number of H868R_07 cells was significantly higher than that of Vector cells. However, the ability of H868R_07 cells to grow in low serum was significantly reduced when cells were treated with rapamycin. As shown in representative photomicrographs at the day 6 incubation time point, H868R_07 cells displayed a spindle-like morphology, grew on the top of each other, and formed foci-like cell clusters, which are typical morphological changes observed in transformed NIH3T3 cells incubated in low serum media (Figure 7B, lower panels). These morphological changes were not observed when H868R_07 cells were treated with rapamycin. Likewise, data from soft agar assays demonstrated that rapamycin treatment significantly diminished the number of colonies formed by H868R_07 cells (Figure 7C). Furthermore, H868R_07 cells no longer formed colonies that were ≥ 100 μm when incubated with rapamycin. PF-4708671 (PF) is a specific inhibitor of S6K1. PF treatment also significantly inhibited the growth of H868R_07 cells in low serum media (1% CS) in comparison with untreated H868R_07 cells (p < 0.01) (Figure 7D). It should be noted that WT_118 cells did not confer a proliferative advantage in low serum media, suggesting the upregulated lipid kinase activity of VPS34 was essential for VPS34 to induce growth in low serum. Consistent with these findings, we also noticed a significant decline in colony formation when H868R_07 cells were incubated with PF as compared with untreated H868R_07 cells (Figure 7E). We further extended our study to investigate whether loss of RheB could affect the ability of H868R_07 cells to grow in low serum and to form colony in soft agar. RheB knockdown was achieved using RheB specific siRNA (Figure 7F). As shown in Figure 7G and 7H, RheB silencing in H868R_07 cells significantly inhibited the growth of these cells in low serum media (Figure 7G) and significantly impaired the ability of these cells to form colonies in soft agar (Figure 7H). Taken together, these findings indicate that VPS34-induced cellular transformation is mediated by mTORC1/S6K1 signaling pathway.

## DISCUSSION

**Figure 8:**
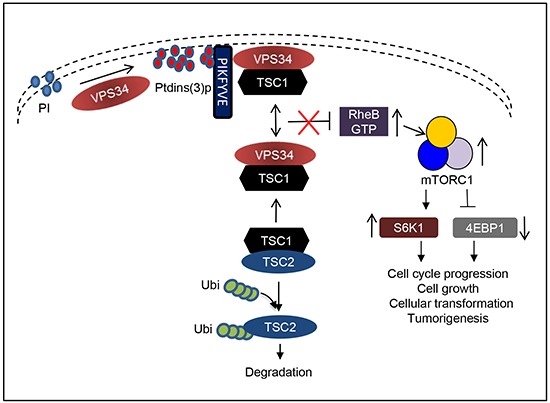
Working model depicting VPS34 regulation of mTOR/S6K1 via recruitment of PIKFYVE and TSC1 to the plasma membrane to mediate TSC2 ubiquitination and degradation and RheB activation Refer to the discussion section for the detailed description.

Class I PI3Ks, which are composed of p110α, the catalytic subunit, and p85, the regulatory subunit, are important in regulating proliferation and tumorigenesis [21]. Mechanisms behind the function of the class I PI3K are well characterized. In cells, p85 binds to p110α and stabilizes p110α to inactivate its kinase activity. Upon growth factor stimulation, p85 binds to phosphorylated tyrosine of receptor via its SH2 domain (Rous sarcoma oncogene homology-2 domain). This binding relieves the inhibition of p110α and mediates the recruitment of p110α to the plasma membrane and activation of its kinase activity to modulate mTOR/S6K1 signaling [21]. Here, we describe a novel mechanism by which VPS34 regulates mTOR/S6K1 via the formation of VPS34/TSC1 protein complex to downregulate TSC2 protein and activate RheB. In our proposed working model (Figure 8), VPS34 binds to TSC1 at the steady state and this interaction is tightly regulated. When VPS34 is overexpressed or activated (i.e., VPS34-H868R), VPS34 increases production of ptdins(3)p at the plasma membrane, which recruits FYVE domain containing protein, PIKFYVE, to ptdins(3)p enriched regions in the plasma membrane where VPS34 forms a protein complex with PIKFYVE and TSC1 (reference 20, Figure 3 and 4). While binding of VPS34 to TSC1 is independent of PIKFYVE, the membrane localization of VPS34, which is at least partially via PIKFYVE, may help stabilize VPS34/TSC1 protein complex at the plasm membrane. This is supported by the data demonstrating that VPS34 kinase dead mutant blocks recruitment of PIKFYVE to the plasma membrane, and therefore interrupted VPS34/TSC1 complex localization at the plasma membrane. The formation of VPS34/TSC1 complex at the plasma membrane disengages TSC2 from the TSC1/TSC2 heterodimer, leading to TSC2 ubiquitination and degradation. Downregulation of TSC2 may reduce the GAP activity of TSC2 and promotes RheB activation, resulting in the activation of mTOR/S6K1. When VPS34 lipid kinase activity is increased by introduction of an H868R mutation, ptdins(3)p production at the plasma membrane is significantly increased, which recruits more PIKFYVE and TSC1 molecules to the ptdins(3)p enriched regions in plasma membrane. This results in the enhanced TSC2 ubiquitination and degradation, leading to increase in RheB to mTORC1/S6K1 signaling, cellular transformation, and tumor formation in mice. These data are consistent with the report by Garami et al, which shows that a loss-of-function mutant in TSC2 GAP domain failed to block RheB activation of S6K1 [34]. The role played by VPS34 in regulating cellular transformation via mTOR/S6K1 pathway is underscored by the fact that downregulation of RheB, mTOR, and S6K1 by RheB specific siRNA, rapamycin and PF-4708671, respectively, inhibits VPS34-mediated cellular transformation.

PIKFYVE targets many cytosolic proteins to ptdins(3)p-enriched membranes and mediates mTOR activation [32, 33] and also plays a role during the oncogenesis [35]. Its lipid product regulates cancer cell migration and invasion [36]. VPS34 is a main source of ptdins(3)p which is required for constitutive PIKFYVE functionality [37]. This information provides the evidence suggesting that PIKFYVE may be a downstream effector of VPS34 to mediate cellular transformation and tumorigenesis that may be independent of VPS34-mediated TSC1/TSC2 to RheB signaling pathway. By producing more ptdins(3)p, VPS34-H868R may enhance PIKFYVE activity which may also contribute to VPS34-induced cellular transformation.

VPS34 has been studied extensively in the context of endocytic sorting, and has been implicated in regulating nutrient signaling [15, 16, 19, 38]. However, the role of VPS34 in the regulation of cell growth and cellular transformation has not been well studied. Overexpression of the wild-type p110α of class I PI3K can lead to oncogenic transformation in cell culture [27, 39]. Additionally, the cancer-specific mutants of p110α protein are oncogenic both *in vitro* and *in vivo* [28]. The oncogenic activity of p110α cancer-specific mutants is reflected in their enzymatic properties such that the cancer-specific mutants display enhanced lipid kinase activity as compared with the wild-type p110α. Similar to p110α, overexpression of VPS34-WT results in cellular transformation. When VPS34 is engineered with an H868R mutation that significantly enhances the lipid kinase activity, the transformation activity of VPS34 is significantly enhanced, suggesting that the extent of oncogenic transformation induced by VPS34 is correlated with VPS34 lipid kinase activity. Tumors induced by dysregulation of the class I PI3K pathway depend on mTORC1 and are exquisitely sensitive to the mTOR inhibitor rapamycin and its derivatives [28, 40, 41]. Our data demonstrate that the inhibitors of mTORC1 and S6K1, rapamycin and PF-4708671, inhibit VPS34-H868R-induced oncogenic transformation, indicating that mTORC1/S6K1 pathway is necessary for VPS34-induced oncogenic transformation. Although the tumor-specific VPS34 mutations have not been identified, this structural and functional study provides mechanistic insight into the cellular functions of VPS34 in the regulation of oncogenic transformation and important clues for identifying VPS34 cancer-specific mutations.

## MATERIALS AND METHODS

### Materials

NIH3T3 cells and COS-7 cells were purchased from American Type Culture Collection (ATCC) and used within six months after cells were ordered. These cell lines were not authenticated in our laboratory. Rapamycin, 5-Bromo-2′-deoxyuridine (BrdU) and cycloheximide were obtained from Sigma. PF-4708671 was purchased from Selleckchem. MG132 was purchased from CalBiochem. Antibodies directed against Myc-tag (9E10), Flag-tag, and HA-tag were obtained from Covance. Antibodies directed against VPS34 (cat#4263), VPS15 (cat#14580), Beclin 1 (cat#3495), pT389-S6K1 (cat#9234), S6K1 (cat#2708), pT37/46-4EBP1 (cat#2855), 4EBP1 (cat#9644), pS235/236-S6 (cat#4858), S6 (cat#2217), pErk1/2 (cat#4370), Erk1/2 (cat#4695), pS473-Akt(cat#4060), Akt (cat#4685), RheB (cat#13879), TSC1 (cat#6935), TSC2 (cat#4308), BrdU (cat#5292), and Cyclin E (cat#4129) were purchased from Cell Signaling Technology. Fluorescently tagged antibodies were obtained from Invitrogen. The class III PI3K lipid kinase ELISA kit was purchased from Echelon Biosciences. The RheB activation kit was purchased from Abcam. 4-6 weeks old SCID mice were ordered from National Cancer Institute (NCI), NIH (Bethesda, MD).

### Cell culture, plasmids and generation of stable clones

Wild-type pcDNA3-Myc-hVPS34 was a kind gift from Dr. Jonathan Backer of Albert Einstein College of Medicine. The kinase dead VPS34 construct, pcDNA3-Myc-hVPS34-D747N, N748K (designated VPS34-KD), and pJ4HA-VPS34 constructs were generated as described previously [20, 31, 42]. The HA-tagged ubiquitin construct was a kind gift from Drs. Dirk Bohman (the EMBL, Heidelberg, Germany) and Yosef Yarden (Weizmann Institute of Science, Israel). pcDNA3-Myc-VPS34-H868R and pJ4H-VPS34-H868R plasmids were generated by Genewiz. NIH3T3 cells stably expressing pJ4H empty vector, VPS34-WT or VPS34-H868R were generated by co-transfecting pcDNA3.1 vector plus pJ4H empty vector, pJ4H-VPS34 or pJ4H-VPS34-H868R as described previously. Stable cell line selection was achieved using G418. PIKFYVE siRNA, RheB siRNA and control siRNA were obtained from GE Dharmacon. RNAi transfections were performed using Lipofectamine 3000 (Invitrogen) according to manufacturer's protocol.

### Lipid kinase assay

VPS34 lipid kinase activity was assessed using a class III PI3K ELISA kit (Echelon Biosciences) according to the manufacturer's instruction. This assay is a competitive ELISA in which the signal is inversely proportional to the amount of ptdins(3)p produced. Briefly, COS-7 cells were seeded in 6-well plates at a density of 1×10^5^ cells per well and transiently transfected with empty vector or plasmids encoding VPS34 or VPS34 mutants. Cells were harvested 48h post transfection and WCL were subjected to immunoprecipitation. The immunoprecipitated VPS34 proteins bound on beads were used for the kinase assay. The absorbance was read at 450nm on an ELISA plate reader. OD values were plotted against Log ptdins(3)p values to generate a standard curve using a sigmoidal dose response. Ptdins(3)p production (pmol) for each enzymatic reaction was determined by interpolation from the standard curve.

### RheB activation assay

The RheB activation assay was performed using a RheB activation kit (ab173243, Abcam) according to the manufacturer's protocol. Briefly, Vector, WT_118 and H868R_07 cells were cultured in 10 cm dishes until they reached 90% confluence (~10^7^ cells). Following washing with ice-cold PBS, the cells were lysed in 0.5 ml 1X ice-cold Assay/Lysis Buffer. Whole cell lysates (WCL) obtained from Vector, WT_118, and H868R_07 were subjected to the assay. WCL were incubated with 1 μL anti-active RheB mouse monoclonal antibody and 20 μL protein A/G agarose bead slurry at 4°C for 1 hour with gentle agitation. Beads were pelleted by centrifugation, washed 3 times with Lysis Buffer and then subjected to SDS-PAGE analysis. RheB was detected by Western blot analysis using a rabbit antibody directed against RheB-GTP. The samples of positive and negative controls were generated according to manufacturer's protocol.

### TSC2 ubiquitination and degradation assay

NIH3T3 cells were seeded overnight and then transiently co-transfected with plasmid encoding HA-tagged ubiquitin, along with Myc-VPS34-WT, Myc-VPS34-H868R or Vector using Lipofectin and PLUS reagent (Life Technologies). 48h post-transfection, WCL were subjected to immunoprecipitations using anti-TSC2 or IgG control antibody. Ubiquitinated endogenous TSC2 was detected by Western blot with anti-HA antibody. The immunoprecipitated endogenous TSC2 was detected by Western blot using anti-TSC2 antibody for assessing TSC2 degradation. The protein levels of Myc-tagged VPS34-WT and Myc-tagged VPS34-H868R in the WCL were detected by Western blot using anti-Myc antibody. For the detection of Flag-tagged TSC2 ubiquitination, COS7 cells were transiently co-transfected with plasmids encoding empty vector, VPS34-WT or VPS34-H868R plus Flag-TSC2 and HA-ubiquitin. 48h post-transfection, Flag-tagged TSC2 was immunoprecipitated from WCL using an anti-Flag antibody. Ubiquitinated Flag-TSC2 was detected by anti-HA antibody. Cycloheximide protein chase experiment was performed to monitor degradation of TSC2. Cells stably expressing vector and VPS-34 (H868R_07) were incubated with cycloheximide at 50 μg/ml for 2, 4 and 6h or left untreated. After incubation, WCL was collected and western blotting was performed to detect the levels of TSC2 using anti-TSC2 antibody. TSC2 accumulation assay was performed using MG132, a potent, reversible and membrane-permeable inhibitor of proteasome. Stably expressing H868R_07 cells were treated with MG132 at 5μM for 24h or left untreated. WCL was subjected to western blot analysis to detect TSC2 expression levels.

### BrdU incorporation cell cycle assay

The experiments were performed according to manufacturer's instructions (Cell Signaling Technology). For flow cytometric cell cycle analysis of BrdU incorporation, one million cells were harvested by centrifugation, and incubated with fresh serum free media containing BrdU (0.03 mg/ml) for 30 min at 37°C. Cells were rinsed, fixed in cold 70% ethanol, incubated with 1.5M HCl for 30 min at room temperature and rinsed with PBS three times. Cells were blocked in incubation buffer (0.5% BSA in PBS) for 10 min, and incubated with mouse anti-BrdU primary antibody at a 1:200 dilution in incubation buffer for 1 hr at room temperature. Cells were washed with PBS and incubated with anti-mouse Alexa Fluor 488 conjugated secondary antibody for 30 min. Cells were washed again and re-suspended in 1 ml PBS, and analyzed on a BD FACS Calibur flow cytometer. Data are presented in the form of histogram plots and quantified as percentage of BrdU-positive cells that are present in S phase of cell cycle.

### Statistical analysis

GraphPad Prism and Microsoft Excel software were used for statistical studies. Statistical significance was determined by Student's t-test (*, p < 0.05; **, p < 0.01). Data are expressed as mean ± SEM.

### Animal experiments

All animal experiments were performed in accordance with animal protocol #WO-2013-08, which was approved by the United States Food and Drug Administration (FDA) Center for Biologics Evaluation and Research (CBER), Institutional Animal Care and Use Committee, in accordance with the U.S. Public Health Service Policy on Humane Care and Use of Laboratory Animals. Four-to-six weeks old male severely compromised immune-deficient (SCID) mice purchased from the National Cancer Institute (NCI) were allowed at least one week to acclimatize to the animal facility prior to the initiation of experiments. Mice were randomly assigned into 3 experimental groups (Vector, VPS34-WT and VPS34-H868R). Stably expressing cell lines were cultured in DMEM media supplemented with 10% calf serum (CS). At 80% confluence, cells were harvested by trypsinization, washed twice with PBS and counted. Cells were then centrifuged at 1000 rpm for 5 min and cell pellets were re-suspended in 400 μL PBS. 1×10^7^ cells were injected subcutaneously into each dorsal site of SCID mice using a 27-gauge needle on syringe. Tumors were allowed to grow 4 weeks. At the end of the experiments, tumors were excised from mice and fixed in 10% neutral buffered formalin (Sigma). Fixed samples were sent to Histoserve, Inc (Germantown, MD) for paraffin sectioning and hematoxylin and eosin (H&E) staining for histopathological evaluation of tumors. H&E stained slides were scanned using panoramic MIDI scanner (Caliper Life Sciences). The images were acquired at a 20X magnification, and relevant areas were converted into a TIF format for the generation of figures. Stained tissue sections were analyzed by a board-certified veterinary pathologist.

## References

[1] Schmelzle T, Hall MN (2000). TOR, a central controller of cell growth. Cell.

[2] Gingras AC, Raught B, Sonenberg N (2001). Regulation of translation initiation by FRAP/mTOR. Genes Dev.

[3] Fingar DC, Richardson CJ, Tee AR, Cheatham L, Tsou C, Blenis J (2004). mTOR controls cell cycle progression through its cell growth effectors S6K1 and 4E-BP1/eukaryotic translation initiation factor 4E. Mol Cell Biol.

[4] Cuyàs E, Corominas-Faja B, Joven J, Menendez JA, Noguchi E, Gadaleta MC (2014). Cell Cycle Regulation by the Nutrient-Sensing Mammalian Target of Rapamycin (mTOR) Pathway. Cell Cycle Control.

[5] Dunlop EA, Tee AR (2009). Mammalian target of rapamycin complex 1: Signalling inputs, substrates and feedback mechanisms. Cellular Signaling.

[6] Huang J, Manning BD (2008). The TSC1-TSC2 complex: a molecular switchboard controlling cell growth. Biochem J.

[7] Castro AF, Rebhun JF, Clark GJ, Quilliam LA (2003). Rheb binds tuberous sclerosis complex 2 (TSC2) and promotes S6 kinase activation in a rapamycin- and farnesylation- dependent manner. J Biol Chem.

[8] Inoki K, Li Y, Xu T, Guan KL (2003). Rheb GTPase is a direct target of TSC2 GAP activity and regulates mTOR signaling. Genes Dev.

[9] Zhang Y, Gao X, Saucedo LJ, Ru B, Edgar BA, Pan D (2003). Rheb is a direct target of the tuberous sclerosis tumour suppressor proteins. Nat Cell Biol.

[10] Inoki K, Li Y, Zhu T, Wu J, Guan KL (2002). TSC2 is phosphorylated and inhibited by Akt and suppresses mTOR signalling. Nat Cell Biol.

[11] Jin F, Wienecke R, Xiao GH, Maize JC, DeClue JE, Yeung RS (1996). Suppression of tumourigenicity by the wild-type tuberous sclerosis 2 (*Tsc2*) gene and its C-terminal region. Proc Natl Acad Sci USA.

[12] Benvenuto G, Li S, Brown SJ, Braverman R, Vass WC, Cheadle JP, Halley DJ, Sampson JR, Wienecke R, DeClue JE (2000). The tuberous sclerosis-1 (*TSC1*) gene product hamartin suppresses cell growth and augments the expression of the TSC2 product tuberin by inhibiting its ubiquitination. Oncogene.

[13] Chong-Kopera H, Inoki K, Li Y, Zhu T, Garcia-Gonzalo FR, Rosa JL, Guan KL (2006). TSC1 stabilizes TSC2 by inhibiting the interaction between TSC2 and the HERC1 ubiquitin ligase. J Biol Chem.

[14] Hay N, Sonenberg N (2014). Upstream and downstream of mTOR. Genes Dev.

[15] Byfield MP, Murray JT, Backer JM (2005). hVps34 is a nutrient-regulated lipid kinase required for activation of p70 S6 kinase. J Biol Chem.

[16] Nobukuni T, Joaquin M, Roccio M, Dann SG, Kim SY, Gulati P, Byfield MP, Backer JM, Natt F, Bos JL, Zwartkruis FJ, Thomas G (2005). Amino acids mediate mTOR/raptor signaling through activation of class 3 phosphatidylinositol 3OH-kinase. Proc Natl Acad Sci USA.

[17] Nobukuni T, Kozma SC, Thomas G (2007). hVps34, an ancient player, enters a growing game: mTOR complex 1/S6K1 signaling. Curr Opin Cell Biol.

[18] Dann SG, Selvaraj A, Thomas G (2007). mTOR complex 1-6K1 signaling: at the crossroads of obesity, diabetes and cancer. Trends Mol Med.

[19] Backer JM (2008). The regulation and function of class III PI3Ks: novel roles for Vps34. Biochem J.

[20] Hirsch DS, Shen Y, Dokmanovic M, Yu J, Mohan N, Elzarrad MK, Wu WJ (2014). Insulin activation of vacuolar protein sorting 34 mediates localized phosphatidylinositol 3-phosphate production at lamellipodia and activation of mTOR/S6K1. Cell Signal.

[21] Bader AG, Kang S, Zhao L, Vogt PK (2005). Oncogenic PI3K deregulates transcription and translation. Nat Rev Cancer.

[22] Samuels Y, Wang Z, Bardelli A, Silliman N, Ptak J, Szabo S, Yan H, Gazdar A, Powell SM, Riggins GJ, Willson JK, Markowitz S, Kinzler KW (2004). High frequency of mutations of the PIK3CA gene in human cancers. Science.

[23] Broderick DK, Di C, Parrett TJ, Samuels YR, Cummins JM, McLendon RE, Fults DW, Velculescu VE, Bigner DD, Yan H (2004). Mutations of PIK3CA in anaplastic oligodendrogliomas, high-grade astrocytomas, and medulloblastomas. Cancer Res.

[24] Bachman KE, Argani P, Samuels Y, Silliman N, Ptak J, Szabo S, Konishi H, Karakas B, Blair BG, Lin C, Peters BA, Velculescu VE, Park BH (2004). The PIK3CA gene is mutated with high frequency in human breast cancers. Cancer Biol Ther.

[25] Engelman JA, Chen L, Tan X, Crosby K, Guimaraes AR, Upadhyay R, Maira M, McNamara K, Perera SA, Song Y, Chirieac LR, Kaur R, Lightbown A (2008). Effective use of PI3K and MEK inhibitors to treat mutant Kras G12D and PIK3CA H1047R murine lung cancers. Nat Med.

[26] Lee JW, Soung YH, Kim SY, Lee HW, Park WS, Nam SW, Kim SH, Lee JY, Yoo NJ, Lee SH (2005). PIK3CA gene is frequently mutated in breast carcinomas and hepatocellular carcinomas. Oncogene.

[27] Kang S, Bader AG, Vogt PK (2004). Phosphatidylinositol 3-kinase mutations identified in human cancer are oncogenic. Proc Natl Acad Sci USA.

[28] Bader AG, Kang S, Vogt PK (2006). Cancer-specific mutations in PIK3CA are oncogenic in vivo. Proc Natl Acad Sci USA.

[29] Watanabe S, Kazumichi S, Okazaki Y, Tonogi M, TanakA Y, Yamane G (2009). Activation of PI3K-AKT pathway in oral epithelial dysplasia and early cancer of tongue. Bull Tokyo Dent Coll.

[30] Hirsch DS, Shen Y, Dokmanovic M, Wu WJ (2010). pp60c-Src phosphorylates and activates vacuolar protein sorting 34 to mediate cellular transformation. Cancer Res.

[31] Wu WJ, Erickson JW, Lin L, Cerione RA (2000). The γ-subunit of the coatomer complex binds Cdc42 to mediate transformation. Nature.

[32] Kutateladze TG (2007). Mechanistic similarities in docking of the FYVE and PX domains to phosphatidylinositol 3-phosphate containing membranes. Prog Lipid Res.

[33] Bridges D, Ma JT, Park S, Inoki K, Weisman LS, Saltiel AR (2012). Phosphatidylinositol 3,5-bisphosphate plays a role in the activation and subcellular localization of mechanistic target of rapamycin 1. Mol Biol Cell.

[34] Garami A, Zwartkruis FJT, Nobukuni T, Joaquin M, Roccio M, Stocker H, Kozma SC, Hafen E, Bos JL, Thomas G (2003). Insulin activation of Rheb, a mediator of mTOR/S6K/4E-BP signaling, is inhibited by TSC1 and 2. Mol Cell.

[35] Coronas S, Lagarrigue F, Ramel D, Chicanne G, Delsol G, Payrastre B, Tronchère H (2008). Elevated levels of PtdIns5P in NPM-ALK transformed cells: implication of PIKfyve. Biochem Biophys Res Commun.

[36] Oppelt A, Haugsten EM, Zech T, Danielsen HE, Sveen A, Lobert VH, Skotheim RI, Wesche J (2014). PIKfyve, MTMR3 and their product PtdIns5P regulate cancer cell migration and invasion through activation of Rac1. Biochem J.

[37] Ikonomov OC, Sbrissa D, Venkatareddy M, Tisdale E, Garg P, Shisheva A (2015). Class III PI 3-kinase is the main source of Ptdlns3P substrate and membrane recruitment signal for PIKfyve constitutive function in podocyte endomembrane homeostasis. Biochim Biophys Acta.

[38] Jaber N, Zong WX (2013). Class III PI3K Vps34: essential roles in autophagy, endocytosis, and heart and liver function. Ann NY Acad Sci.

[39] Aoki M, Schetter C, Himly M, Batista O, Chang HW, Vogt PK (2000). The catalytic subunit of phosphoinositide 3-kinase: requirements for oncogenicity. J Biol Chem.

[40] Neshat MS, Mellinghoff IK, Tran C, Stiles B, Thomas G, Petersen R, Frost P, Gibbons JJ, Wu H, Sawyers CL (2001). Enhanced sensitivity of PTEN-deficient tumors to inhibition of FRAP/mTOR. Proc Natl Acad Sci USA.

[41] Podsypanina K, Lee RT, Politis C, Hennessy I, Crane A, Puc J, Neshat M, Wang H, Yang L, Gibbons J, Frost P, Dreisbach V, Blenis J (2001). An inhibitor of mTOR reduces neoplasia and normalizes p70/S6 kinase activity in Pten+/− mice. Proc Natl Acad Sci USA.

[42] Wu WJ, Tu SS, Cerione RA (2003). Activated Cdc42 sequesters c-Cbl and prevents EGF receptor degradation. Cell.

